# The effects of submaximal exercise and cold exposure on blood coagulation parameters in coronary artery disease patients

**DOI:** 10.1186/s12872-021-01907-9

**Published:** 2021-02-16

**Authors:** Karri Parkkila, Rasmus I. P. Valtonen, Leena Hiltunen, Heidi E. Hintsala, Jouni J. K. Jaakkola, Tiina M. Ikäheimo

**Affiliations:** 1 Center for Environmental and Respiratory Health Research (CERH), University of Oulu, P.O. Box 5000, 90014 Oulu, Finland; 2 Medical Research Center, University of Oulu, Oulu University Hospital, Oulu, Finland; 3 Centria University of Applied Sciences, Kokkola, Finland; 4 Biocenter Oulu, University of Oulu, Oulu, Finland; 5 Department of Hemostasis, Finnish Red Cross Blood Service, Helsinki, Finland; 6 Present Address: Hemostasis and Platelet Laboratory, Fimlab Laboratoriot Oy Ltd, Vantaa, Finland

**Keywords:** Exercise, Cold, Coagulation, Coronary artery disease

## Abstract

**Background:**

Both exercise and cold exposure increase blood coagulation potential but their combined effects are not known. The purpose of the present study was to assess blood coagulation factors in response to submaximal exercise in the cold environment among patients with stable coronary artery disease (CAD).

**Methods:**

Sixteen men (61.1 ± 7.1 years) with stable CAD participated in three 30-min experimental conditions (seated rest in − 15 °C and exercise in both + 22 °C and − 15 °C) in random order. The employed exercise consisted of brisk walking (66–69% of maximal heart rate). Factor VII (FVII), fibrinogen, D-dimer and von Willebrand factor (vWF) were analyzed from blood samples obtained before, immediately and one hour after each experiment.

**Results:**

On average, FVII activity (95% confidence interval, CI) was 123 (108–143) %, 123 (106–140) %, 121 (103–139) % (baseline, recovery 1, recovery 2), fibrinogen concentration (95% CI) 3.81 (3.49–4.12) g/l, 3.71 (3.34–4.08) g/l, 3.65 (3.26–4.05) g/l, D-dimer concentration (95% CI) 0.42 (0.28–0.56) µg/ml, 0.42 (0.29-.55) µg/ml and 0.39 (0.29–0.49) µg/ml, and vWF activity (95% CI) 184 (135–232) %, 170 (128–212) % and 173 (129–217) % after exercise in the cold. Average FVII activity varied from 122 to 123%, fibrinogen concentration from 3.71 to 3.75 g/l, D-dimer concentration from 0.35 to 0.51 µg/ml and von Willebrand factor activity from 168 to 175% immediately after each three experimental condition.

**Conclusions:**

Our findings suggest that submaximal lower body exercise carried out in a cold environment does not significantly affect blood coagulation parameters among patients with stable CAD.

## Background

Regular exercise improves cardiovascular health, decreases mortality and reduces the risk of developing a cardiovascular disease through mechanisms, such as lowered resting blood pressure, reduced progression of atherosclerotic disease and improved blood oxygen-carrying capacity [1]. In coronary artery disease (CAD) patients, exercise-based cardiac rehabilitation decreases cardiovascular mortality and hospital admissions [2]. Physical activity for cardiac rehabilitation should consist of regularly performed, moderate intensity endurance training, lasting at least 30 min at a time [3].

Irrespective of the beneficial effects, physical activity acts as an important external trigger for acute myocardial infarction [4]. In addition, high levels of coagulation parameters, such as factor VII (FVII), fibrinogen, von Willebrand factor (vWF), as well as low fibrinolytic activity have been associated with cardiovascular diseases [5–9]. Exercise has been shown to increase blood coagulation potential in both healthy people [10] and cardiovascular disease patients [11] and the elevated coagulation potential is usually accompanied with increased fibrinolytic activity [10, 12]. These changes in hemostatic profile, however, depend on the intensity and duration of the physical activity. Especially high-intensity or prolonged exercise increases the blood coagulation potential, while moderate exercise or shorter exercise bouts have a lesser impact on the hemostatic profile [10, 13]. Most adverse cardiovascular events, such as unstable angina, myocardial infarction, cerebrovascular accidents and sudden cardiac deaths, occur due to an occlusive blood clot [14]. Therefore, acute hemostatic responses to physical exercise are of great clinical significance.

Based on recent systematic reviews, cold temperature increases cardiovascular strain and may elevate coagulation and fibrinolytic activity [15, 16]. There is evidence that short-term decrease in ambient temperature increases hospitalizations for ischemic heart diseases [17] and that cardiovascular mortality follows a seasonal pattern where mortality rates are higher in colder seasons and climates [18]. Likewise, an epidemiological study conducted in the United Kingdom, showed that blood coagulation parameters peaked during the coldest time of the year [19]. However, there are only a few previous controlled experiments exploring the effects of acute cold exposure on blood coagulation profile. In a controlled experiment, Keatinge et al. [20] reported increases in erythrocyte, platelet and inflammatory cell count, as well as increased blood overall viscosity during mild surface cooling. Therefore, cold exposure can increase the risk of arterial thrombosis and partially contribute to the higher occurrence of cold-related cardiovascular events [21].

As both exercise and cold exposure may separately increase the coagulation potential, it is important to understand their joint effect. Nagelkirk et al. [22] showed in a controlled experiment that maximal exercise performed in a cold environment increased blood coagulation potential more compared with that performed at a neutral temperature. These effects may be especially pertinent among CAD patients whose blood coagulation potential is already elevated because of the disease itself [23]. However, there are no previous studies exploring the joint effects of submaximal exercise and cold exposure on blood coagulation parameters in CAD patients.

The purpose of the current study was to elucidate the effects of acute submaximal lower body exercise and whole-body cold exposure on blood coagulation parameters among CAD patients. It was hypothesized that cold temperature and submaximal exercise occurring in combination would increase the coagulation parameters more than expected from their Independent effects.

## Methods

### Study population

The study population, design and safety protocols have been described in detail earlier [24, 25]. An experienced cardiologist identified participants with CAD and myocardial infarction from the patient registry of the Oulu University Hospital (2014–2016) and inquired their willingness to participate in the study. The patients of this study were treated at the University Hospital during the acute phase of their myocardial infarction which included angiography of coronary arteries and placement of stents. The inclusion criteria included diagnosed CAD (Canadian Cardiac Society class I-II) and a non-ST-elevation myocardial infarction at least 3 months before the experiments. The exclusion criteria were the following: Canadian Cardiac Society class III-IV, previous myocardial infarction less than 3 months before the recruitment, chronic atrial fibrillation, claudication, unstable angina pectoris, left ventricular ejection fraction < 40%, a history of coronary artery bypass crafting, pacemaker, serious ECG anomalies at rest, presence of physician-diagnosed asthma or diabetes and current smoking. A complete set of coagulation parameters were obtained from a total of sixteen men (age: 61.1 ± 7.1 years, body mass index: 28.3 ± 4.9 kg/m^2^) and their characteristics are presented in Table 1. The study was approved by the Ethics Committee of Oulu University Hospital District and the study is registered in the Clinical Trials (no. NCT02855905 date 04/08/2016). The participants received both oral and written information of the study, and they signed an informed consent. Full details of the research protocol can be found in the Additional file 1.

**Table 1:** Characteristics of the study group

Variables	(n) 16
Age, years	61.1 ± 7.1
BMI, kg/m^2^	28.3 ± 4.9
BF, %	23.2 ± 7.4
Peak V_O2_, ml⋅kg^−1^⋅min^−1^	30.8 ± 4.8
SBP, mmHg	123 ± 24
DBP, mmHg	75 ± 13
*Hypertension*	
Yes	10 (63%)
No	6 (38%)
*Medications*	
Aspirin	14 (88%)
β-blockers	9 (56%)
Statins	12 (75%)
ADP receptor antagonist	6 (38%)
ACE-inhibitors	11 (69%)
ATR-blocker	2 (13%)
Calcium channel blocker	1 (6%)
*How do you find your current health status?*	
Excellent	3 (19%)
Quite good	6 (38%)
Average	7 (44%)
Quite poor	0 (0%)
Very poor	0 (0%)
Alcohol within 12 months	
Yes	15 (94%)
No	1 (6%)
*Pensioner*	
Yes	9 (56%)
No	7 (44%)
*How demanding is/was your work physically?*	
My work is mainly done sitting	7 (44%)
I walk quite much in my work	4 (25%)
I have to walk and lift much	4 (25%)
My work represents heavy manual labor	1 (6%)
*How much do you exercise and stress yourself physically in your leisure time?*	
Never	2 (13%)
Rarely	10 (63%)
Often	3 (19%)
Very often	1 (6%)

Values are the number of the patients (% within the group) or means ± SD. BMI, body mass index; BF, body fat percentage; Peak V̇_O2_, estimated (3.5 × MET, where MET is metabolic equivalent of task) symptom-limited maximal oxygen uptake; SBP, systolic blood pressure; DBP, diastolic blood pressure; ADP, adenosine‐diphosphate; ACE, angiotensin-converting enzyme; ATR, angiotensin receptor

### Study design

In the current study, each subject participated in a total of three different 30-min experimental conditions in random order. The order of these was based on balloting performed by the researchers. These experiments included resting (sitting) in a cold environment (− 15 °C, wind 1.0 m/s), and exercising both in a neutral (+ 22 °C, wind 1.0 m/s) and cold environment (− 15 °C, wind 1.0 m/s). The employed exercise consisted of brisk walking for 30 min on a treadmill. Clinical exercise tests were performed prior to the experiments to asses maximal exercise capacity [25] and the results were used to calculate an individually based walking speed for the experimental condition that represented moderate intensity exercise (65–70% of the individual heart rate maximum). The chosen exercise type and intensity represents physical exercise recommendations for CAD patients [3]. The cold exposure mimics everyday winter conditions in the northern climate. While resting in the cold, the participants wore full winter clothing (clothing insulation value 2.13 clo) and the cold exposure was targeted mainly to their face and airways. During exercise in the cold, the participants removed their insulated trousers and jacket (clothing insulation 1.88 clo). They used less clothing (0.75 clo) during experiments in neutral conditions.

The experiments were conducted in winter 2015–2016 during the same time of the day for each participant and there was at least one week in between the experiments. The subjects were instructed to avoid heavy exercise 24 h before, alcohol 48 h before and caffeine related beverages 2 h before the experiments. Prior to the experiments, the participants completed questionnaires inquiring of their health and lifestyle, physical fitness, current health status and exposure to cold at work or during their leisure time. In addition, their body composition was assessed by bioimpedance measurements (InBody720 Biospace, Seoul, Korea). The participants were equipped with 10 skin temperature thermistors and ECG electrodes. After the instrumentation, baseline measurements were carried out in a seated position in a climate chamber with neutral temperature (+ 22 °C). The experimental trials (rest or exercise for 30 min) were conducted in an adjacent climate chamber with temperature set either to − 15 °C or + 22 °C. After each experimental condition, the participants were followed up in neutral conditions for 60 min.

### Measured parameters

#### Blood coagulation parameters

The primary outcome of interest of the present study are the blood coagulation parameters. A total of three blood samples from each participant were drawn during each experiment: after 15 min of sitting in neutral conditions (baseline), as well as immediately (recovery 1) and 60 min after (recovery 2) each experimental condition. Blood was collected by venipuncture using 20-gauge needle into tubes containing 3.2% trisodium citrate (0.109 M, 0.1vol). The first tube was discarded to avoid tissue factor contamination of the sample. Plasma was prepared within an hour after the last sampling of each participant by centrifugation at 2500 g for 15 min in room temperature. Prepared plasma was stored in aliquots at − 80 °C until analyzed.

Fibrinogen was measured by using Clauss method (STA-Liquid Fib, Diagnostica Stago). D-dimer was measured by using immune-turbidimeric assay (STA®-Liatest® D-Di Plus, Diagnostica Stago). Factor VII activity was measured using prothrombin time based one-stage assay (Stago Deficient VII, Diagnostica Stago). VWF activity was measured by using turbidimetric assay (Innovance® VWF Ac, Siemens, OPHL). All analyzes were done using automated coagulation analyzer (STA-R Max® analyzer, Diagnostica Stago). When analyzing each variable, all samples of one patient were analyzed at the same time in the same run to avoid inter assay variation. Laboratory personnel were blinded to the participants and experimental conditions.

For each coagulation parameter, the reference ranges of Fimlab hemostasis laboratory were used: FVII activity (78–155%), fibrinogen (2.0–4.0 g/l), D-dimer (< 0.5 µg/ml), and vWF activity (49–205%).

#### Physical strain and thermal responses

Physical strain during exercise was measured by continuous monitoring of heart rate and by inquiring of the perceived exertion at 5-min intervals (Borg scale). Skin temperature was monitored continuously using thermistors (NTC DC95, Digi-Key, Thief River Falls, MN) attached to the right scapula, left cheek, forehead, left calf, right anterior thigh, left index finger, left hand, left forearm, right shoulder and left upper chest. Mean skin temperature (T_sk_) was calculated as follows: tsk = ∑ki*tski = [0.07*forehead + 0.175*right scapula + 0.175*left upper chest + 0.07*right arm + 0.07*left arm + 0.05*left hand + 0.19*right anterior thigh + 0.2*left calf] [26]. Subjective thermal sensation was inquired every 5 min using scales of perceptual judgements of personal thermal state [27].

### Statistical methods

A sample size estimation and power analysis (G-Power 3.1.0) was conducted prior to the study which estimated 15 participants to be sufficient to detect statistically significant differences in blood pressure (our primary outcome measure in the broader study) between a warm and cold environment [Power (1-ß err prob), 0.9, Cohen’s effect size 0.8, α err prob 0.05]. The normality assumptions of the dependent variables (FVII, fibrinogen, D-dimer, vWF) was tested using the Shapiro–Wilk test. All parameters except D-dimer were normally distributed, for which a natural logarithmic transformation was used for statistical analyses. Means in coagulation parameters were compared with a repeated measures ANOVA using within-participants condition (cold rest, neutral exercise, cold exercise) and test time (baseline, recovery 1, recovery 2). Statistical analyses were performed with IBM SPSS version 25 for Microsoft Windows and statistical significance was set at P < 0.05.

## Results

Nine of the sixteen participants were pensioners (Table 1). Their physical fitness (mean VO_2peak_ 30.8 ± 4.8 ml/kg/min) was mediocre according to reference values for peak oxygen uptake [28]. All the participants rated their health status as being moderate or better. Despite of this, 12 persons (75%) reported being only rarely or never physically active during their leisure time. The average time elapsed from myocardial infarction was 17 ± 6 months. Nine (56%) of the participants had a single-vessel, six (38%) a double-vessel and one (6%) a triple-vessel disease. Six participants had one stent, five had two stents and the rest had 3 to 5 stents. The left ventricular ejection fraction of the participants was on average 60 ± 11% and varied from 41 to 79%.

### Skin temperature, thermal sensation and level of exercise

The mean skin temperature was 25.7 ± 0.9 °C (rest in the cold), 28.6 ± 0.8 °C (exercise in neutral environment) and 23.3 ± 0.9 °C (exercise in the cold) immediately after each experimental condition. Mean skin temperature was 6.3 ± 1.0 °C lower immediately after exercise in the cold compared to pre-exposure baseline (p < 0.001). Facial skin temperature decreased significantly from 31 ± 0.4 °C to 12 ± 1.3 °C after both rest and exercise in the cold environment (p < 0.001). The average individual whole-body thermal sensation was − 3/cold (rest in the cold), + 2/warm (exercise in neutral conditions) and − 1/slightly cool (exercise in the cold) at the end of each condition. On average, the achieved exercise intensity represented 66% (neutral conditions) and 69% (cold conditions) of the heart rate maximum. The rate of perceived exertion using the Borg scale varied from fairly light to somewhat hard while exercising in neutral or in cold conditions.

### Effect of submaximal exercise in cold ambient temperature on blood coagulation parameters

**Fig. 1:**
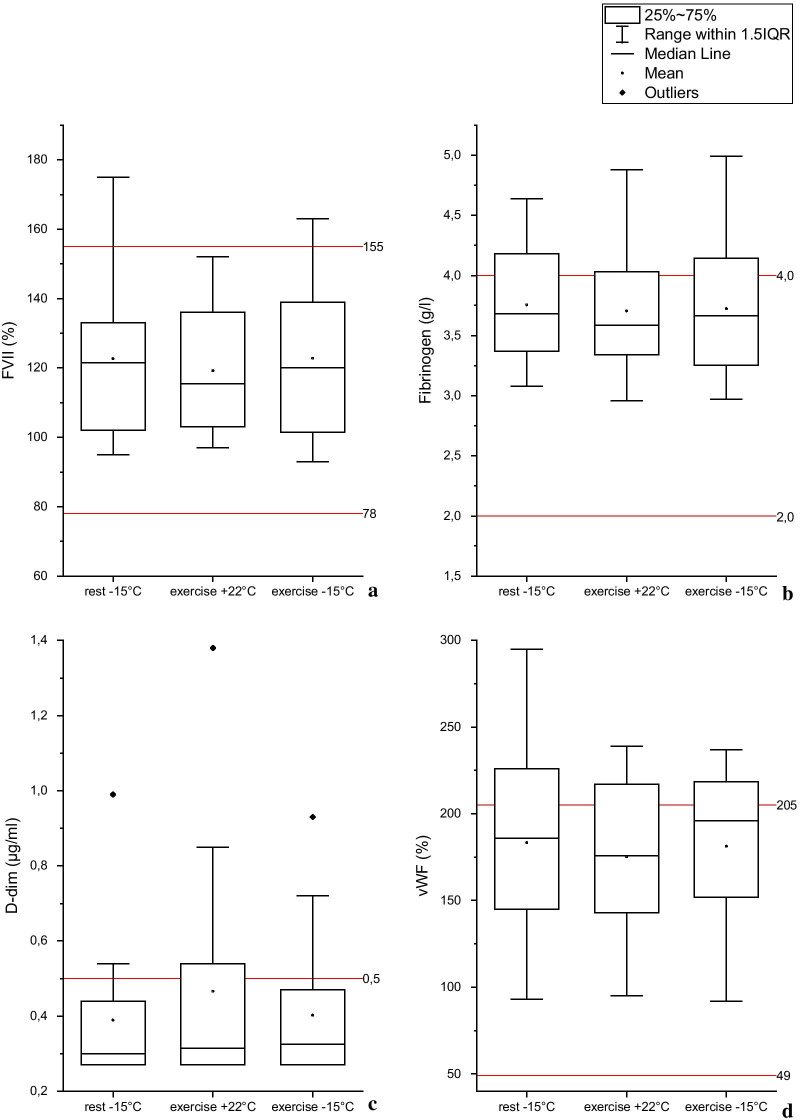
Blood coagulation factors obtained immediately after rest or exercise at either + 22 ºC or − 15 °C among CAD patients

The employed experimental condition and test time showed statistically significant interaction only for FVII, F (4, 32) = 3.201, p = 0.026. Figure 1 represents coagulation parameter values obtained immediately after each experimental condition.

The box plot charts represent blood coagulation factor values immediately after each experimental condition (rest at − 15 °C and exercise at + 22 °C or − 15 °C) in coronary artery disease patients (n = 16). The red lines indicate the reference range for each coagulation factor. (Fig. 1 legend).

#### Factor VII

Factor VII activity (95% CI) was on average 116 (103–130) %, 122 (108–136) %, 126 (108–145) % (baseline, recovery 1, recovery 2) during rest in cold, 126 (106–145) %, 122 (106–137) %, 126 (110–141) % during the exercise in neutral conditions and 123 (108–143) %, 123 (106–140) %, 121 (103–139) % during the exercise in cold. At baseline, nearly all FVII values were in the upper half of the reference range (78–155)%. The effects of condition F (2, 16) = 0.275, p = 0.763 or test time, F (2, 16) = 1.829, p = 0.193 were not significant. Two (13%) of the participants demonstrated FVII levels that exceeded the reference range following rest or exercise in the cold. In total, FVII activity decreased in ten and increased in six participants after the exercise in the cold (Table 2).

**Table 2:** Individual responses in blood coagulation factors immediately after each experimental condition

Rest -15 °C	FVII (%)		Fibrinogen (g/l)		D-dim (µg/ml)		vWF (%)	
Patient	Baseline	Change	Baseline	Change	Baseline	Change	Baseline	Change
1	118	↑ 9.3%	3.85	↑ **4.7%**	0.27	↔	104	↓ 10.6%
2	106	↑ 8.5%	**4.05**	↑ **3.2%**	0.35	↓ 2.9%	186	↑ **17.2%**
3	117	↓ 6.0%	3.35	↓ 2.4%	0.27	↔	185	↓ 6.5%
4	94	↑ 5.3%	3.14	↑ 13.7%	0.27	↑ **88.9%**	177	↓ 1.7%
5	135		3.39		0.27	↔	155	
6	132	↑ 10.6%	3.43	↑ 7.3%	0.36	↓ 25%	109	↓ 12.8%
7	109	↓ 6.4%	3.61	↑ 1.9%	0.29	↔	156	↓ 7.1%
8	114	↑ 8.8%	**4.31**	↑ **7.7%**	**0.66**	↑ **50%**	189	↑ **16.4%**
9	**167**	↑ **4.8%**	3.97	↓ 0.25%	0.27	↔	143	↑ 10.5%
10	136	↑ 5.1%	**4.28**	↓ **2.1%**	0.34	↑ 20.6%	**206**	↑ **10.7%**
11	84	↑ 13.1%	3.49	↓ 1.4%	0.27	↔	**215**	↑ **7.9%**
12	130	↑ 2.3%	3.44	↓ 2.0%	**0.63**	↓ **14.3%**	112	↔
13	101		**5.36**		**0.79**		**271**	
14	126	↑ 2.4%	3.17	↑ 1.6%	0.27	↑ 14.8%	201	↓ 1.5%
15	103	↑ 15.5%	2.96	↑ 4.1%	0.27	↔	**239**	↑ **23.4%**
16	99	↓ 1.0%	**4.2**	↑ **1.4%**	**0.63**	↓ 30.2%	**229**	↓ **1.3%**

Exercise + 22 °C	FVII (%)		Fibrinogen (g/l)		D-dim (µg/ml)		vWF (%)	
Patient	Baseline	Change	Baseline	Change	Baseline	Change	Baseline	Change
1	143	↓ 11.9%	**4.36**	↓ **7.6%**	0.27	↑ 14.8%	99	↓ 4.0%
2	83	↑ 16.9%	3.56	↑ 10.4%	0.29	↑ **121%**	176	↑ 3.4%
3	114	↓ 2.6%	3.24	↓ 2.5%	0.27		175	↓ 16.0%
4	108	↓ 3.7%	3.62	↓ 7.7%	0.37	↑ 13.5%	195	↓ 12.8%
5	145	↓ 2.1%	3.6	↓ 5.6%	0.27	↔	167	↑ 1.8%
6	142	↔	3.82	↓ 1.3%	0.31	↓ 12.9%	96	↑ 1.0%
7	100	↑ 3.0%	3.78	↓ 6.1%	0.37	↓ 18.9%	127	↑ 12.6%
8	–		–		–		–	
9	–		–		–		–	
10	140	↓ 5.7%	**4.43**	↓ **6.5%**	0.42	↓ 23.8%	**214**	↔ 0.0%
11	105	↓ 5.7%	3.33	↓ 1.8%	0.27	↔	**246**	↓ **6.9%**
12	146	↓ 6.8%	3.67	↓ 1.4%	**1.56**	↓ **11.5%**	135	↓ 1.5%
13	108	↓ 4.6%	**4.93**	↓ **1.0%**	0.27	↑ **100%**	**217**	↑ **1.4%**
14	**158**	↓ 3.8%	3.59	↓ 1.7%	0.44	↓ 38.6%	**211**	↓ 6.6%
15	125	↓ 4.0%	3.3	↓ 10.3%	0.27	↑ 51.9%	**252**	↓ **5.2%**
16	101	↑ 0.99%	**4.28**	↑ **0.47%**	**0.72**	↑ **18.1%**	**210**	↑ **3.3%**

Exercise -15 °C	FVII (%)		Fibrinogen (g/l)		D-dim (µg/ml)		vWF (%)	
Patient	Baseline	Change	Baseline	Change	Baseline	Change	Baseline	Change
1	120	↑ 0.8%	**4.08**	↑ **1.5%**	**0.78**	↓ **7.7%**	100	↓ 8.0%
2	124	↓ 4.8%	**4.02**	↑ **3.2%**	0.48	↑ **20.8%**	**208**	↓ **1.4%**
3	118	↓ 2.5%	3.27	↓ 4.6%	0.27	↑ 22.2%	188	↑ 2.1%
4	107	↓ 7.5%	3.27	↓ 9.2%	0.27	↔	167	↓ 0.60%
5	144	↓ 6.9%	3.46	↓ 2.0%	0.3	↓ 10.0%	170	↓ 9.4%
6	**165**	↓ **4.8%**	3.31	↓ 6.0%	0.27	↔	104	↓ 0.96%
7	101	↓ 5.9%	3.93	↓ 3.3%	0.36	↓ 11.1%	135	↑ 66.7%
8	127	↑ 7.9%	**4.11**	↑ **3.6%**	**0.78**	↑ **19.2%**	182	↑ **19.8%**
9	152	↑ **7.2%**	3.85	↓ 0.78%	0.27	↔	178	↓ 15.7%
10	124	↓ 4.0%	**4.5**	↓ **1.8%**	0.33	↑ 6.1%	**260**	↓ 24.6%
11	93	↑ 3.2%	3.59	↓ 3.6%	0.27	↔	**260**	**↓ 9.6%**
12	137	↑ 2.9%	3.75	↓ 8.3%	**0.55**	**↑ 7.3%**	114	↓ 7.9%
13	108	↓ 3.7%	**5.14**	**↓ 2.9%**	0.47	↓ 27.7%	**227**	**↑ 4.4%**
14	150	↓ 1.3%	3.56	↓ 0.84%	0.27	↑ 11.1%	199	↓ 1.5%
15	119	↑ 4.2%	2.99	↓ 0.33%	0.27	↔	**216**	**↑ 1.4%**
16	97	↓ 4.1%	**4.17**	**↓ 3.8%**	**0.53**	↓ 32.1%	**220**	**↓ 5.9%**

Individual responses in blood coagulation factors (FVII, Fibrinogen, D-dim, vWF) immediately after each experimental condition (rest at -15 °C and exercise at + 22 °C or -15 °C) in coronary artery disease patients (n = 16). The relative change in coagulation factors occurring immediately after the intervention compared with baseline is presented. Bolded baseline values and/or relative changes (recovery 1) indicate values that exceed the reference values: FVII, 78–155%; Fibrinogen, 2.0–4.0 g/l; D-dim, < 50 µg/ml; vWF, 49–205%

#### Fibrinogen

Fibrinogen concentration (95% CI) was on average 3.70 (3.38–4.01) g/l, 3.74 (3.41–4.07) g/l, 3.68 (3.39–3.98) g/l (baseline, recovery 1, recovery 2) during rest in the cold, 3.81 (3.47–4.15) g/l, 3.75 (3.45–4.05) g/l, 3.86 (3.47–4.25) g/l during the exercise in neutral conditions and 3.81 (3.49–4.12) g/l, 3.71 (3.34–4.08) g/l, 3.65 (3.26–4.05) g/l during the exercise in cold. The effects of condition F (2, 16) = 1.1, p = 0.357 or test time F (2, 16) = 0.44, p = 0.651 were not significant. At baseline, fibrinogen levels of the participants were in the highest quarter of the reference range (2.0–4.0 g/l). Four (25%) of the participants demonstrated values above the reference range after every experimental condition. Fibrinogen concentrations decreased immediately after exercise in the cold in the majority (81%) of the participants (Table 2).

#### D-Dimer

D-dimer concentration (95% CI) was on average 0.38 (0.26–0.49) µg/ml, 0.35 (0.27–0.42) µg/ml, 0.32 (0.24–0.39) µg/ml (baseline, recovery 1, recovery 2) during rest in the cold, 0.51 (0.18–0.83) µg/ml, 0.51 (0.21–0.81) µg/ml, 0.56 (0.23–0.88) µg/ml during the exercise in neutral conditions and 0.42 (0.28–0.56) µg/ml, 0.42 (0.29–0.55) µg/ml and 0.39 (0.29–0.49) µg/ml during the exercise in cold. The effects of condition F (1.789, 14.309) = 1.850, p = 0.195 or test time F (2, 16) = 0.107, p = 0.9 were not significant. Five (31%) of the participants had values above the reference range (< 50 µg/ml) after the exercise in cold. One patient had D-dimer values almost three-fold above the reference range in neutral conditions: 1.56 µg/ml (baseline), 1.38 µg/ml (recovery 1), and 1.59 µg/ml (recovery 2). In total, D-dimer values increased among six (38%), decreased among five (31%) and did not change in  five (31%) of the participants following exercise in the cold (Table 2).

#### Von Willebrand factor

VWF activity (95% CI) was on average 172 (134–210) %, 175 (129–221) %, 177 (133–222) % (baseline, recovery 1, recovery 2) during the rest in cold, 174 (133–214) %, 168 (128–208) %, 193 (154–232) % during the exercise in neutral conditions and 184 (135–232) %, 170 (128–212) % and 173 (129–217) % during the exercise in cold. The effects of condition F (2, 16) = 0.194, p = 0.826 or test time F (2, 16) = 1.884, p = 0.184 were not significant. Six (38%) of the participants demonstrated values above the reference range (49–205%) following the exercise in cold. The vWF activity of one patient increased significantly from 135% (baseline) to 225% (recovery 1) after exercising in the cold. Compared to others, another patient reacted strongly following exercise in neutral conditions with an increase of vWF activity from 96% (baseline) to 232% (recovery 2). The majority (69%) of participants demonstrated decreased, while 31% demonstrated increased vWF activity immediately after exercising in the cold (Table 2).

## Discussion

This is the first study to examine the independent and joint effects of exercise and cold exposure on coagulation parameters among participants with stable CAD. The novel results of the current study indicate that submaximal lower body exercise performed in cold ambient temperature does not induce significant alterations in blood coagulation parameters acutely. This finding suggests that such conditions would not increase the risk for thrombosis within the studied population.

Exercise is known to activate sympathetic nervous system and increase blood catecholamine levels in an intensity-dependent manner [29]. Sympathetic activation may lead to increased platelet count and activation, as well as increased FVIII activity and vWF release via beta-adrenergic receptor stimulation and endothelial activation [30–33]. VWF has been shown to have an important role in vascular inflammation and thrombus formation [34]. Considering its possible role in atherosclerosis, as well as in predicting cardiovascular events [34, 35], increased vWF release could contribute, in particular, to exercise-induced pathological outcomes. Additionally, high-intensity exercise and stenotic arteries may lead to pathological shear stress on the endothelial wall resulting in attenuated nitric oxide production, endothelial dysfunction and a pro-coagulant microenvironment [36, 37]. Previous studies have shown exercise-induced increases in platelet counts and function, blood coagulation markers and fibrinolysis [38–40]. These changes in hemostatic profile, however, are influenced particularly by the intensity of the exercise [13]. For instance, Menzel & Hilberg [10] showed that exercise at 100% of individual anaerobic threshold (IAT) leads to significantly greater increase in factor VIII and thrombin-antithrombin complex (TAT) compared to 80% IAT. Moreover, Cadroy et al. [40] found an increased thrombotic tendency only after intense exercise and Wang et al. [41] showed even decreased platelet aggregation and adhesion immediately after moderate-intensity exercise of 30 min. Exercise in the current study consisted of brisk walking on a treadmill for 30 min and the achieved intensity represented the physical activity guidelines for cardiac rehabilitation [3]. The results presented in the present paper support the previous findings [10, 13] suggesting that moderate intensity exercise exerts minor or no effects on blood coagulation parameters.

There are only few experimental studies investigating how acute cold exposure affects blood coagulation parameters. Cold-induced vasoconstriction and elevated blood pressure [15, 16] might increase shear stress on the endothelial wall, which could manifest as increased coagulation potential through platelet aggregation, as well as vWF unfolding and self-association [33, 42]. An experimental study with eight healthy 18–25 years old participants showed that mild surface cooling for six hours (+ 24 °C, air speed 10 m/s) increased erythrocyte, thrombocyte and leucocyte counts, as well as overall blood viscosity [20]. It should be noted, that these changes had barely started within the first hour and most of the effects were observed after six hours of monitoring. In contrast, Nagelkirk et al. [22] reported elevated coagulation potential (increased thrombin-antithrombin concentration) after only 15 min of rest in a cold (5 or 8 °C) environment. An epidemiologic study by Woodhouse [19] reported increased fibrinogen and FVII activity during winter, but these responses were related to various inflammation markers and self-reported cough, but not to ambient temperature. In accordance with the results of the current study, Mercer et al. [44] did not detect any significant changes in fibrinogen or FVII following a mild exposure to cold stress (+ 11 °C) even though they had a much longer follow-up period (48 h) compared to current study (60 min). Therefore, regardless that cold activation of FVII has been reported in blood samples [45], it seems that it does not occur in people exposed to cold. The deviating findings in the discussed studies likely relate to the duration and intensity of cold exposure, examined coagulation parameters, as well as timing of the measurements.

Exercise-induced shear stress combined with cold temperature may lead to impaired regulation of vascular tonus [36, 46] and activation of vWF [33, 34] which further potentiates blood coagulation. Moreover, atherosclerosis and CAD itself could result in elevated coagulation potential [23]. These factors might explain the higher occurrence of sudden cardiac deaths triggered by winter-time activities among CAD patients compared to other cardiovascular disease patients [47]. Currently, there is only one previous study exploring the joint effects of exercise and cold temperature on blood coagulation parameters. Nagelkirk et al. [22] studied the impact of maximal exercise, performed at different environmental temperatures (5–8 °C, 20 °C or 30 °C), on blood coagulation potential among healthy young (19–35 years old) participants. They observed that maximal exercise increased thrombin-antithrombin concentrations, and that cold temperature further potentiated this effect. In contrast, no marked effects of either submaximal exercise itself, or an added effect of cold exposure (− 15 °C) were found in the current study for fibrinogen, FVII, vWF or D-Dimer levels among CAD patients. However, a few subjects demonstrated notable increases in D-dimer and/or vWF concentrations after exercise in neutral or cold conditions. Based on the sensitivity analyses of the current study, these participants did not differ from the rest of the study population in terms of age, BMI, perceived level of exertion, thermal sensations or medication. The deviating results between the studies likely relate to the differences in exercise intensity, studied populations and examined coagulation parameters. At baseline, the participants in the present study demonstrated fibrinogen and vWF values which were in the upper parts of the reference ranges. This observation agrees with the previous studies suggesting that coagulation potential could be elevated with CAD itself [23] as well as due to aging [48].

### Perspectives and significance

Exercise and cold exposure increase cardiac strain in patients with cardiovascular diseases [15, 16]. In fact, physical activity is a common external trigger for acute cardiovascular events [4]. Additionally, cold temperature separately increases hospitalizations and mortality for cardio- and cerebrovascular diseases [17, 49]. Elevated coagulation potential related to cold exposure [20] and exercise [12] independently and together [22], as well as the CAD-related hypercoagulable state [23] could manifest as thrombotic events. Winter-time physical activities, such as skiing and snow shoveling, can therefore expose especially CAD patients to sudden cardiac deaths [47]. However, at the same time regular year-round exercise plays a crucial role as a rehabilitative treatment for CAD patients. Exercise-based cardiac rehabilitation reduces cardiovascular mortality, hospital admissions, and may decrease health care costs compared to non-exercising control participants [2].

This is the first study exploring the joint effects of submaximal exercise and cold exposure on blood coagulation parameters of stable CAD patients. The results presented here suggest that coagulation is not acutely altered after submaximal lower body exercise performed in cold conditions in patients with stable CAD. Furthermore, Valtonen et al. [25] reported that submaximal exercise performed in cold conditions increased cardiac workload but did not cause myocardial ischemia or impaired endothelial function among stable CAD patients [50]. Hence, year-round health-enhancing exercise appears to be a safe treatment for patients with stable CAD even with recurrent cold ambient temperatures. The current study provides information for health care professionals and can be utilized to encourage patients to engage in regular physical activities. By doing so, the overall health of cardiovascular patients and their functional capabilities are expected to be maintained or promoted.

## Strengths and limitations

The strengths of the present study include precisely controlled thermal exposure mimicking common winter conditions in northern countries. In addition, the employed exercise represented physical activity guidelines for cardiovascular patients [3]. The comprehensive crossover study design eliminated possible order effects and reduced confounding from factors other than those related to CAD.

There are some limitations related to the present study. The study participants represents only a relatively healthy population of men with stable CAD. Acclimatization to winter conditions could have affected baseline levels of coagulation parameters, and possibly the magnitude of their responses. Finally, for safety reasons the participants were not instructed to withdraw their medications which could have affected the results. Although, by not withdrawing the medications it was possible to evaluate the coagulation parameter responses of individuals who are being treated for CAD, rather than responses without any preventative medication.

## Conclusions

To conclude, no significant changes in blood coagulation parameters (FVII, fibrinogen, D-dimer, vWF) were observed in stable CAD patients in response to submaximal lower body exercise performed in cold ambient temperature. Future studies involving different study populations (e.g. more advanced state of the disease or other cardiovascular diseases) and exercise intensities are needed to further elucidate these interactions to help us achieve a more comprehensive understanding on the subject.

## Data Availability

All data generated or analyzed and presented in this study are included in this published article.

## Abbreviations

CAD: Coronary artery disease
FVII: Factor VII
vWF: Von Willebrand factor
ECG: Electrocardiogram
TAT: Thrombin-antithrombin
IAT: Individual anaerobic threshold
BMI: Body mass index

## References

[1] Nystoriak MA, Bhatnagar A (2018). Cardiovascular effects and benefits of exercise. Front Cardiovasc Med.

[2] Anderson L, Oldridge N, Thompson DR, Zwisler AD, Rees K, Martin N, Taylor RS (2016). Exercise-based cardiac rehabilitation for coronary heart disease: cochrane systematic review and meta-analysis. J Am Coll Cardiol.

[3] Association E, of Cardiovascular Prevention and Rehabilitation Committee for Science Guidelines, EACPR, Corrà, U., Piepoli, M. F., Carré, F., Heuschmann, P., Hoffmann, U., Verschuren, M., Halcox, J., Document Reviewers, Giannuzzi, P., Saner, H., Wood, D., Piepoli, M. F., Corrà, U., Benzer, W., Bjarnason-Wehrens, B., Dendale, P., Gaita, D., McGee, H., … Schmid, J. P.  (2010). Secondary prevention through cardiac rehabilitation: physical activity counselling and exercise training: key components of the position paper from the Cardiac Rehabilitation Section of the European Association of Cardiovascular Prevention and Rehabilitation. Eur Heart J.

[4] Culić V, Eterović D, Mirić D (2005). Meta-analysis of possible external triggers of acute myocardial infarction. Int J Cardiol.

[5] Meade TW, Mellows S, Brozovic M, Miller GJ, Chakrabarti RR, North WR, Haines AP, Stirling Y, Imeson JD, Thompson SG (1986). Haemostatic function and ischaemic heart disease: principal results of the Northwick Park Heart Study. Lancet (London, England).

[6] Meade TW, Ruddock V, Stirling Y, Chakrabarti R, Miller GJ (1993). Fibrinolytic activity, clotting factors, and long-term incidence of ischaemic heart disease in the Northwick Park Heart Study. Lancet (London, England).

[7] Spiel AO, Gilbert JC, Jilma B (2008). von Willebrand factor in cardiovascular disease: focus on acute coronary syndromes. Circulation.

[8] Lowe G (2011). Can haemostatic factors predict atherothrombosis?. Intern Emerg Med.

[9] Lacey B, Herrington WG, Preiss D, Lewington S, Armitage J (2017). The role of emerging risk factors in cardiovascular outcomes. Curr Atheroscl Rep.

[10] Menzel K, Hilberg T (2011). Blood coagulation and fibrinolysis in healthy, untrained subjects: effects of different exercise intensities controlled by individual anaerobic threshold. Eur J Appl Physiol.

[11] Mustonen P, Lepäntalo M, Lassila R (1998). Physical exertion induces thrombin formation and fibrin degradation in patients with peripheral atherosclerosis. Arterioscler Thromb Vasc Biol.

[12] Womack, C. J., Nagelkirk, P. R., & Coughlin, A. M. (2003). Exercise-induced changes in coagulation and fibrinolysis in healthy populations and patients with cardiovascular disease. *Sports medicine (Auckland, N.Z.)*, *33*(11), 795–807. https://doi.org/10.2165/00007256-200333110-00002 10.2165/00007256-200333110-00002 12959620

[13] Posthuma JJ, van der Meijden PE, Ten Cate H, Spronk HM (2015). Short- and Long-term exercise induced alterations in haemostasis: a review of the literature. Blood Rev.

[14] Ardissino, D., Merlini, P. A., Eisenberg, P. R., Kottke-Marchant, K., Crenshaw, B. S., & Granger, C. B. (1998). Coagulation markers and outcomes in acute coronary syndromes. *American heart journal*, *136*(4 Pt 2 Su), S7–S18. https://doi.org/10.1053/hj.1998.v136.93436 10.1053/hj.1998.v136.93436 9778084

[15] Manou-Stathopoulou V, Goodwin CD, Patterson T, Redwood SR, Marber MS, Williams RP (2015). The effects of cold and exercise on the cardiovascular system. Heart (British Cardiac Society).

[16] Ikäheimo T. M. (2018). Cardiovascular diseases, cold exposure and exercise. *Temperature (Austin, Tex.)*, *5*(2), 123–146. https://doi.org/10.1080/23328940.2017.1414014 10.1080/23328940.2017.1414014 PMC6204981 30377633

[17] Lin S, Soim A, Gleason KA, Hwang SA (2016). Association between low temperature during winter season and hospitalizations for ischemic heart diseases in New York State. J Environ Health.

[18] Marti-Soler H, Gonseth S, Gubelmann C, Stringhini S, Bovet P, Chen PC, Wojtyniak B, Paccaud F, Tsai DH, Zdrojewski T, Marques-Vidal P (2014). Seasonal variation of overall and cardiovascular mortality: a study in 19 countries from different geographic locations. PLoS ONE.

[19] Woodhouse PR, Khaw KT, Plummer M, Foley A, Meade TW (1994). Seasonal variations of plasma fibrinogen and factor VII activity in the elderly: winter infections and death from cardiovascular disease. Lancet (London, England).

[20] Keatinge WR, Coleshaw SR, Cotter F, Mattock M, Murphy M, Chelliah R (1984). Increases in platelet and red cell counts, blood viscosity, and arterial pressure during mild surface cooling: factors in mortality from coronary and cerebral thrombosis in winter. Br Med J (Clinical research ed).

[21] Sartini C, Barry S, Wannamethee SG, Whincup PH, Lennon L, Ford I, Morris RW (2016). Effect of cold spells and their modifiers on cardiovascular disease events: Evidence from two prospective studies. Int J Cardiol.

[22] Nagelkirk PR, Hogan KB, Hoare JM (2012). Ambient temperature affects thrombotic potential at rest and following exercise. Thromb Res.

[23] Tosi F, Micaglio R, Sandri M, Castagna A, Minguzzi D, Stefanoni F, Chiariello C, Franzese I, Luciani GB, Faggian G, Girelli D, Olivieri O, Martinelli N (2017). Increased plasma thrombin potential is associated with stable coronary artery disease: An angiographically-controlled study. Thromb Res.

[24] Ikäheimo TM, Länsitie M, Valtonen RIP, Hintsala HE, Ryti NRI, Perkiömäki J, Mäntysaari M, Hautala AJ, Jaakkola JJK (2019). Good safety practice in a randomized controlled trial (CadColdEx) involving increased cardiac workload in patients with coronary artery disease. BMC Cardiovasc Disord.

[25] Valtonen, R.I.P, Kiviniemi, A., Hintsala, H. E., Ryti, N.R.I, Kenttä, T., Huikuri, H. V., Perkiömäki, J., Crandall, C., van Marken Lichtenbelt, W., Alén, M., Rintamäki, H., Mäntysaari, M., Hautala, A., Jaakkola, J.J.K., & Ikäheimo, T. M. (2018). Cardiovascular responses to cold and submaximal exercise in patients with coronary artery disease. *American journal of physiology. Regulatory, integrative and comparative physiology*, *315*(4), R768–R776. https://doi.org/10.1152/ajpregu.00069.2018 10.1152/ajpregu.00069.2018 29975565

[26] International Organization for Standardization. ISO 9886. Evaluation of thermal strain by physiological measurements.[Standard revision ISO 9886:2004: https://www.iso.org/standard/17767.html.] 1991.

[27] International Organization for Standardization. ISO 10551:1995. Ergonomics of the thermal environment. Assessment of the influence of the thermal environment using subjective judgement scales. 1995.

[28] Rapp D, Scharhag J, Wagenpfeil S, Scholl J (2018). Reference values for peak oxygen uptake: cross-sectional analysis of cycle ergometry-based cardiopulmonary exercise tests of 10 090 adult German volunteers from the Prevention First Registry. BMJ open.

[29] Zouhal, H., Jacob, C., Delamarche, P., & Gratas-Delamarche, A. (2008). Catecholamines and the effects of exercise, training and gender. Sports medicine (Auckland, N.Z.), 38(5), 401–423. https://doi.org/10.2165/00007256-200838050-00004 10.2165/00007256-200838050-00004 18416594

[30] von Känel R, Dimsdale JE (2000). Effects of sympathetic activation by adrenergic infusions on hemostasis in vivo. Eur J Haematol.

[31] Ali-Saleh, M., Sarig, G., Ablin, J. N., Brenner, B., & Jacob, G. (2016). Inhalation of a Short-Acting β2-Adrenoreceptor Agonist Induces a Hypercoagulable State in Healthy Subjects. *PloS* *one*, *11*(7), e0158652. https://doi.org/10.1371/journal.pone.0158652 10.1371/journal.pone.0158652 PMC4933351 27379911

[33] Chen J, Chung DW (2018). Inflammation, von Willebrand factor, and ADAMTS13. Blood.

[34] Gragnano F, Sperlongano S, Golia E (2017). The role of von Willebrand factor in vascular inflammation: from pathogenesis to targeted therapy. Med Inflamm.

[35] Gragnano F, Golia E, Natale F (2017). Von Willebrand factor and cardiovascular disease: from a biochemical marker to an attractive therapeutic target. Curr Vasc Pharmacol.

[36] Wang YX, Liu HB, Li PS, Yuan WX, Liu B, Liu ST, Qin KR (2018). ROS and NO dynamics in endothelial cells exposed to exercise-induced wall shear stress. Cell Mol Bioeng.

[37] Rana A, Westein E, Niego B, Hagemeyer CE (2019). Shear-dependent platelet aggregation: mechanisms and therapeutic opportunities. Front Cardiovasc Med.

[38] Wang JS (2004). Intense exercise increases shear-induced platelet aggregation in men through enhancement of von Willbrand factor binding, glycoprotein IIb/IIIa activation, and P-selectin expression on platelets. Eur J Appl Physiol.

[39] Sumann G, Fries D, Griesmacher A, Falkensammer G, Klingler A, Koller A, Streif W, Greie S, Schobersberger B, Schobersberger W (2007). Blood coagulation activation and fibrinolysis during a downhill marathon run. Blood Coagul Fibrinol.

[40] Huskens D, Roest M, Remijn JA, Konings J, Kremers RM, Bloemen S, Schurgers E, Selmeczi A, Kelchtermans H, van Meel R, Meex SJ, Kleinegris MC, de Groot PG, Urbanus RT, Ninivaggi M, de Laat B (2016). Strenuous exercise induces a hyperreactive rebalanced haemostatic state that is more pronounced in men. Thromb Haemost.

[40] Cadroy, Y., Pillard, F., Sakariassen, K. S., Thalamas, C., Boneu, B., & Riviere, D. (2002). Strenuous but not moderate exercise increases the thrombotic tendency in healthy sedentary male volunteers. *Journal of applied physiology (Bethesda, Md. : 1985)*, *93*(3), 829–833. https://doi.org/10.1152/japplphysiol.00206.2002 10.1152/japplphysiol.00206.2002 12183474

[41] Wang JS, Jen CJ, Kung HC, Lin LJ, Hsiue TR, Chen HI (1994). Different effects of strenuous exercise and moderate exercise on platelet function in men. Circulation.

[42] Ruggeri ZM, Orje JN, Habermann R, Federici AB, Reininger AJ (2006). Activation-independent platelet adhesion and aggregation under elevated shear stress. Blood.

[44] Mercer JB, Osterud B, Tveita T (1999). The effect of short-term cold exposure on risk factors for cardiovascular disease. Thromb Res.

[45] Kim YA, Lewandrowski KB, Lucien FA, Van Cott EM (2018). The effects of transport temperature and time on routine and specialized coagulation assays. Blood Coagul Fibrinol.

[46] Isa BM, K., Kawasaki, N., Ueyama, K., Sumii, T., & Kudo, S.  (2011). Effects of cold exposure and shear stress on endothelial nitric oxide synthase activation. Biochem Biophys Res Commun.

[47] Toukola T, Hookana E, Junttila J, Kaikkonen K, Tikkanen J, Perkiömäki J, Kortelainen ML, Huikuri HV (2015). Sudden cardiac death during physical exercise: Characteristics of victims and autopsy findings. Ann Med.

[48] Franchini M (2006). Hemostasis and aging. Critical reviews in oncology/hematology.

[49] Bunker A, Wildenhain J, Vandenbergh A, Henschke N, Rocklöv J, Hajat S, Sauerborn R (2016). Effects of air temperature on climate-sensitive mortality and morbidity outcomes in the elderly; a systematic review and meta-analysis of epidemiological evidence. EBioMedicine.

[50] Valtonen RIP, Ikäheimo TM, Hintsala HE, Ryti NRI, Hautala A, Perkiömäki JS, Crandall CG, Mäntysaari M, Jaakkola JJK, Kiviniemi AM (2020). Endothelial function in response to exercise in the cold in patients with coronary artery disease. Clin Physiol Funct Imaging.

